# State of readiness: National health insurance implementation in hospitals, Gauteng province

**DOI:** 10.4102/curationis.v48i1.2689

**Published:** 2025-07-31

**Authors:** Aluwani D. Mudzweda, Thembi V. Simbeni, Ntlogeleng M. Mogale

**Affiliations:** 1 Department of Advance Nursing Sciences, Faculty of Health Sciences, University of Venda, Thohoyandou, South Africa; 2 Department of Public Health, School of Health Care Sciences, Sefako Makgatho Health Sciences University, Pretoria, South Africa

**Keywords:** healthcare practitioners, universal health coverage, National Health Insurance, state of readiness, South Africa

## Abstract

**Background:**

Most nations are implementing the National Health Insurance (NHI) programme to achieve universal health coverage (UHC). In South Africa, the government is working to ensure everyone has access to healthcare and achieve the UHC goal. Challenges in healthcare facilities include inadequate infrastructure, a lack of personnel and a lack of equipment. Researchers have concentrated on the healthcare practitioner’s awareness of the implementation of NHI compared to their state of readiness even though there have been few studies on the readiness for NHI implementation in South Africa.

**Objectives:**

The study explored the opportunities and challenges for healthcare facilities in the readiness for the implementation of NHI.

**Method:**

A non-experimental, quantitative, descriptive, cross-sectional design was employed in this study. The researcher utilised a questionnaire with closed and open-ended questions to obtain information about the state of readiness. The Microsoft Excel spreadsheet was imported to STATA 17 SE software for data analysis.

**Results:**

In total, 352 healthcare practitioners (HCPs) were recruited with a response rate of 93%. The HCPs (36.2%) were skilled and well-trained to function under NHI in their facilities. The main challenges in health facilities were shortage of staff (20.3%), followed by infrastructure (16.3%).

**Conclusion:**

This study suggests that there is a need for training and information sharing on the NHI policy.

**Contribution:**

The study indicates that challenges related to the healthcare system and the success of NHI, among others, are poor infrastructure, shortage of personnel and inadequate resources such as supply.

## Introduction

The NHI is a healthcare financing system that was designed to pool funds to actively purchase and provide access to quality, affordable personal healthcare services for all South Africans based on their health needs, irrespective of their socioeconomic status (Department of Health 2017). The Office of Health Standards and Compliance (OHSC) was established to ensure that hospitals render quality health services following applicable norms and standards (Department of Health 2015). Therefore, the state of readiness of the South African health facilities will be monitored by the OHSC through the Ideal Hospital Realisation Framework assessment.

The preparation for the National Health Insurance (NHI) required certain interventions such as scaling up the Ideal Clinic Model, infrastructure improvement across the health sectors and implementation of the World Health Organization’s (WHO) Workload Indicators for Staffing Needs (WISN) tool. The implementation of NHI is in phases, with Phase 1 being implemented between 2012 and 2017, focused on piloting various health systems’ strengthening interventions at the primary healthcare level to ensure that the health system operated effectively. These interventions included the Ward-based Primary Healthcare Outreach Teams, Ideal Clinic Realisation and Maintenance model, Integrated School Health Programme, Centralised Chronic Medicine Dispensing and Distribution, District Clinical Specialist Teams, Health Patients Registration System, Stock Visibility System, Contracting of general practitioners, Workload Indicator for Staffing Needs and Infrastructure projects. Phase 2 (2017–2022) concentrated on the creation of systems and procedures to ensure that the NHI Fund is operated and administered effectively, and Phase 3 (2021–2025) brings in mandatory NHI prepayments, contracting for authorised private hospitals and specialised services, as well as the finalisation and implementation of the *Medical Schemes Act* (Mukwena & Manyisa 2022).

Several challenges have been noted in Gauteng province, South Africa, such as poor infrastructure, patient-staff ratio issues and limited resources which compromise quality patient care. Several authors have indicated that the challenges plaguing the public healthcare sector such as understaffing, inadequate equipment and shortage of healthcare resources do not inspire confidence in the successful implementation of NHI (Mathew & Mash 2019; Molokomme, Seekoe & Goon 2018). According to a review done by Chuene and Kgarose (2023), another problem impeding the implementation of the NHI in the healthcare system is related to human resources. This has been evidenced by the unbalanced distribution of workload in public facilities.

According to Hajrah, Ahmad and Sakka (2018), in South Konewa Regency, Indonesia, the healthcare facilities are ready for the operationalisation of the NHI programme. In Africa, countries such as Ghana, Nigeria, Rwanda, Kenya and Mali have commenced with NHI programme as a key strategy for universal health coverage (UHC) (Mathew & Mash 2019). Research has shown that Ghana successfully implemented the NHI Scheme for over 15 years (Christmals & Aidam 2020). Studies that were conducted in South Africa focused more on the healthcare practitioner’s (HCP) awareness of the implementation of NHI and less on readiness for the implementation thereof (Mabuza et al. 2018). As a result, the state of readiness of the healthcare facilities was not thoroughly investigated. In this study, the authors aimed to investigate healthcare facilities’ readiness for the implementation of NHI in Sedibeng district, Gauteng province.

## Research methods and design

A non-experimental, quantitative, descriptive, cross-sectional design was employed in this study. The study was conducted in three public hospitals in the Sedibeng district of Gauteng province. Sedibeng district is a rural area, surrounded by informal settlements. The study subjects comprised of HCPs, that is nurses, doctors and allied HCPs employed in those public hospitals, and are approximately 1877 in number.

### Sample size and sampling

Cochrane’s formula for sample size calculation was used based on a 95% confidence level, 5% margin of error and 50% distribution rate. A minimum sample size of 320 was derived and a buffer of 10% was subsequently added resulting in a sample size of 352. All HCPs such as doctors, nurses and allied practitioners working at Sedibeng public hospitals were included in the study. Stratified sampling was used where each potential respondent got a fair representation in the sample. The study sample size was 326, with 49 doctors, 193 nurses and 84 allied personnel.

### Data collection tool, procedure and description of variables

The original questionnaire was a 24-item self-administered tool, developed from literature and validated by a purposefully selected panel of four experts, and was used to collect data. The questionnaire was developed and administered in English, as this was the expected language understood by all HCPs. It comprised of two sections, namely sociodemographic information and the state of NHI implementation readiness. The questionnaire had open and closed-ended questions and following a pre-test with minor modifications, the questionnaire was distributed to 352 eligible HCPs, recruited mainly during tea and lunch breaks. Data were collected by the researchers with the assistance of a trained researcher assistant. The study purpose, respondent’s rights and confidentiality were explained, and consent was obtained from the respondents before completing the questionnaire. On average, one interview took about 10 min to complete. After each interview, each questionnaire was checked for correctness and completeness.

The following sociodemographic information was obtained and these included age, gender, highest level of education, status (nurse, doctor, or allied health practitioner) and years in service. The state of readiness was assessed using four closed-ended questions and one open-ended question. The closed-ended questions were measured on a 3-point Likert scale from ‘disagree’, ‘unsure’ and ‘agree’, and these were coded as ‘1’, ‘2’ and ‘3’, respectively. These questions assessed HCPs’ skills and training, service provision, human resource capacity and infrastructure related to NHI and its implementation. The Likert scale questions assessing the state of readiness had a scale reliability coefficient (Cronbach alpha) of 0.82. The perceived state of NHI implementation readiness among HCPs’ in healthcare facilities was considered positive based on the overall average mean score closer to 3 and vice versa.

### Data analysis

Data were captured and cleaned using Microsoft Excel prior to importation into STATA 17 SE for analysis. Descriptive statistics were used to analyse data. Frequency distribution was used to analyse categorical variables, and continuous variables were analysed using summary statistics. The results from categorical variables are presented as frequency and percentages in tables and graphs, while continuous variables are presented as means and standard deviation. The questions on readiness were also analysed descriptively, and the mean score for each question was analysed and presented with corresponding standard deviation. The overall mean score was calculated from the four questions and presented with corresponding standard deviations. The overall average mean score closer to 3 implies a positive perceived state of readiness. Content analysis was used to analyse the open-ended question where data were categorised, coded and analysed using frequency distribution.

### Ethical considerations

Ethical approval to conduct this study was obtained from the Sefako Makgatho University Research Ethics Committee (SMUREC) (reference number SMUREC/H/166/2022:PG). Permission to conduct the study was sought from the National Department of Health (NDoH). In addition, the Sedibeng District Research Committee provided additional approval and permission to conduct the study in the selected public hospitals. The researcher also sought permission to conduct the study at the selected facilities from the facility managers or Chief Executive Officers (CEOs). The study details were explained to the respondents and a signed informed consent was obtained before the study commenced. All coronavirus disease 2019 (COVID-19) regulations were adhered to by using sanitisers, wearing face masks and maintaining social distancing.

## Results

### Demographic characteristics of healthcare practitioners

A total of 352 questionnaires were distributed among doctors, nurses and allied personnel, of which, only 326 questionnaires were completed and returned, giving a response rate of 93%. All 326 questionnaires were analysed for demographic characteristics and study objectives.

Table 1 shows the demographic characteristics of the HCPs. The predominant age category of HCPs was between 23–33 years old constituting 34.7% (*n* = 113) of the respondents. Most of the respondents (34.1%, *n* = 111) had a bachelor’s degree, followed by those with diplomas (25.2%. *n* = 82). Nurses made up 59.2% of the respondents (*n* = 193), followed by allied workers at 25.8% (*n* = 84). While 36.0% (*n* = 117) of HCPs had less than 5 years of experience in the field, 37.1% (*n* = 121) had experience between 6 and 15 years.

**TABLE 1:** Demographic characteristics of the healthcare practitioners.

Variables	Frequency	%
**Age categories (years)**		
< 22	4	1.23
23–33	113	34.66
34–44	96	29.45
45–55	81	24.85
> 65	32	9.81
Total	326	100.00
**Gender**		
Female	250	76.69
Male	76	23.31
Total	326	100.00
**Educational level**		
Certificate course	68	20.86
Diploma	82	25.15
Bachelor’s degree	111	34.05
Postgraduate diploma	47	14.42
Master’s degree	12	3.68
Doctoral degree	1	0.31
Others	5	1.53
Total	326	100.00
**Status**		
Allied	84	25.77
Doctors	49	15.03
Nurses	193	59.20
Total	326	100.00
**Years of service**		
< 5	117	35.89
6–15	121	37.12
16–25	51	15.64
26–35	26	7.98
36–45	11	3.37
Total	326	100.00

### Healthcare practitioners self-reported state of readiness of National Health Insurance implementation

**FIGURE 1:**
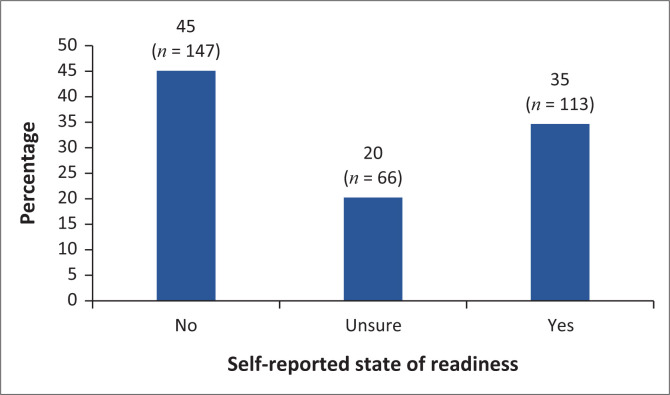
Self-reported state of readiness among healthcare practitioners in the implementation of National Health Insurance in their facility.

Most of the HCPs (45.1%, *n* = 147), when asked whether they were prepared for the implementation of NHI in their facilities, indicated that they were prepared, while 34.7% (*n* = 113) reported not being prepared and 20.3% were unsure (Figure 1).

Table 2 shows the results of HCPs who indicated that their facilities are not prepared for the implementation of NHI. Staff shortage (20.3%; *n* = 66), followed by a lack of infrastructure (16.3%; *n* = 53) were the main reasons why HCPs felt that facilities were not ready to implement NHI. Furthermore, 12.0% (*n* = 40) of HCPs indicated a lack of training, knowledge and skills in facilities, and a similar proportion highlighted poor communication on the NHI policy. About 6% of HCPs (*n* = 18) believed that there are no adequate funds for the implementation of NHI, and therefore this will have serious financial implications for the taxpayers as well as its sustainability.

**TABLE 2:** Reasons for lack of NHI implementation preparedness by HCPs.

Variable	Frequency	%
Lack of training, knowledge, and skills	40	12.27
Staff shortage	66	20.25
Lack of equipment	36	11.04
Inadequate infrastructure	53	16.25
Lack of funds and cost implications	18	5.52
Lack of proper communication of NHI policy	40	12.27

NHI, National Health Insurance; HCPs, healthcare practitioners.

Table 3 shows the perceived state of readiness of HCPs regarding the implementation of NHI in their facilities. Only 36.2% (*n* = 118) of the respondents felt that HCPs in their facilities have skills and are well-trained to function under NHI, while the majority disagreed or were uncertain. In terms of the readiness of their facilities to provide the required NHI services in the future, majority (51.53%, *n* = 168) of the respondents felt their facilities would not be ready. A high proportion of HCPs (67.8%, *n* = 221) felt that their facilities did not have adequate human resources to implement the NHI. Furthermore, (66.6%, *n* = 217) of the respondents also felt that the necessary infrastructure to roll out NHI in their facilities is lacking. The overall mean score of 1.66 (standard deviation [s.d.] = 0.77) for the perceived state of readiness of NHI implementation is very low, indicative of low levels of confidence in the ability of healthcare facilities to implement NHI.

**TABLE 3:** Perceived state of readiness for the implementation of National Health Insurance.

Variables	Disagree		Uncertain		Agree		Mean	s.d.
	*n*	%	*n*	%	*n*	%		
1. Healthcare practitioners are skilled and well-trained to function under NHI in this facility.	109	33.44	99	30.37	118	36.20	2.07	0.83
2. Our facility will be ready to provide the services required when NHI is implemented.	168	51.53	88	26.99	70	21.47	1.70	0.80
3. There is an adequate human resource in my facility for the implementation of NHI.	221	67.79	61	18.71	44	13.50	1.46	0.72
4. There is necessary infrastructure to roll out NHI in our public hospitals.	217	66.56	61	18.71	48	14.72	1.48	0.73

Note: Overall mean score: mean = 1.66; s.d. = 0.77.

s.d., standard deviation; NHI, National Health Insurance.

## Discussion

The study found that more females than males are working in the public healthcare sector, and this imbalance is witnessed across different health professions (Tandwa & Dhai 2020). The policy papers that led to the Draft NHI Bill, the Green Paper, Draft White and White Papers, illustrate the need for NHI in South Africa (Analytics 2019). The successful implementation of NHI hinges on the readiness of both HCPs and facilities; however, significant challenges exist that raise concerns about this preparedness. In this study, HCPs report a lack of adequate training and knowledge about the NHI policies, coupled with poor communication channels. As far back as 2018, a study conducted in Tshwane highlighted poor levels of awareness of NHI among HCPs suggesting that any awareness efforts towards NHI had not taken effect among HCPs (Mabuza et al. 2018). The skills and information gaps need urgent attention to ensure that HCPs are equipped to navigate the complexities of the new system. Transparent communication and stakeholder involvement throughout the entire NHI implementation process are also important (Gaqavu 2018; Michel et al. 2020; Naidoo, Suleman & Bangalee 2024).

In this study, concerns about a lack of and potential mismanagement of funds, as well as perceived corruption are seen as significant barriers to the successful implementation and administration of NHI. These perceptions are often fuelled by media reports that tend to inadvertently erode public trust in health institutions (Molokomme et al. 2018). While some studies suggest a lack of preparedness among public sector HCPs for NHI implementation, contrasting findings exist (Mndzebele & Matsi 2016). Given that HCPs are central to driving NHI, adequate training and skills development are crucial to ensure their effective functioning within the new system. National Health Insurance aims to promote equity, accessibility and utilisation of health services across both the public and private sectors, moving towards a unified national healthcare system and ultimately contributing to UHC and equal access for all citizens of South Africa (Chuene & Kgarose 2023; Matahela, Adekola & Mavhandu-Mudzusi 2023).

Several key factors were highlighted in this study by HCPs as potential impediments to NHI implementation, including inadequate infrastructure, shortage of personnel and equipment. These broader systemic issues such as shortage of human resources, inadequate resources and infrastructural limitations, threaten the effective implementation of NHI and highlight the need for comprehensive and multifaceted interventions (Mathew & Mash 2019; Molokomme et al. 2018). Therefore, addressing human resource shortages is paramount, with several authors advocating for extensive training, skills development and strategic deployment of HCPs (Chuene & Kgarose 2023; Matahela et al. 2023). It is believed that the poor state of population health, due in part to inefficiencies in human resources, will be greatly improved through NHI implementation (Pauw 2022).

### Strengths and limitations

The findings of this study contributed to the existing knowledge on the implementation of NHI. It provides an understanding of the challenges that healthcare facilities will have to manage when implementing NHI. The outcomes of this study could provide valuable information to the South African Department of Health on the state of readiness of the healthcare facilities in order to improve the implementation of NHI going forward.

One of the limitations of this study is that only one district in Gauteng was sampled, and therefore these findings may not represent all districts in South Africa. In addition, the study did not include any private healthcare facilities. Furthermore, there is little literature on similar studies done in Southern Africa. Therefore, it is important for other studies to be carried out in this area, extending the research projects to different provinces of South Africa to obtain a broader view of the healthcare facilities’ state of readiness regarding NHI implementation. Moreover, the study used quantitative approach and most probably a mixed method approach would have yielded rich data.

### Recommendations

Effective communication and consultation before policy development with all HCPs is crucial. That can be achieved through training, workshops and awareness campaigns. To guarantee that there is no shortage of human resources, more healthcare professionals need to be trained. Furthermore, the expedited accreditation of nursing education institutions to allow for the training of more nurses in South Africa by SANC, as well as the accreditation of speciality courses to guarantee that nurses are knowledgeable and competent should also be considered. To effectively tackle the ongoing shortage of healthcare practitioners, especially nurses, the regulatory body should consider increasing the number of nurses accepted per intake at accredited training facilities. This strategic move would ensure an adequate supply of nurses to meet the community’s healthcare needs. To guarantee the availability of high-quality healthcare, the government needs to ensure that there are enough resources, including supplies and adequate infrastructures. Finally, the monitoring and evaluation of NHI implementation must be an ongoing process to proactively identify challenges and ensure timely, effective resolutions.

## Conclusion

This study highlights significant gaps between human resources and infrastructure preparedness for NHI implementation within the Sedibeng public health sector. Despite the positive perception towards NHI among HCPs, the current state of the facilities and human resources poses a substantial challenge to its effective implementation. Therefore, targeted interventions are urgently required, including comprehensive training programmes for HCPs and substantial investment in infrastructural improvements. Furthermore, robust and targeted information dissemination regarding NHI policy is crucial to bridge the gap between policy intent and practical implementation.
